# Diagnostic Accuracy of a Machine Learning-Derived Appendicitis Score in Children: A Multicenter Validation Study

**DOI:** 10.3390/children12070937

**Published:** 2025-07-16

**Authors:** Emrah Aydın, Taha Eren Sarnıç, İnan Utku Türkmen, Narmina Khanmammadova, Ufuk Ateş, Mustafa Onur Öztan, Tamer Sekmenli, Necip Fazıl Aras, Tülin Öztaş, Ali Yalçınkaya, Murat Özbek, Deniz Gökçe, Hatice Sonay Yalçın Cömert, Osman Uzunlu, Aliye Kandırıcı, Nazile Ertürk, Alev Süzen, Fatih Akova, Mehmet Paşaoğlu, Egemen Eroğlu, Gülnur Göllü Bahadır, Ahmet Murat Çakmak, Salim Bilici, Ramazan Karabulut, Mustafa İmamoğlu, Haluk Sarıhan, Süleyman Cüneyt Karakuş

**Affiliations:** 1 Department of Pediatric Surgery, Tekirdağ Namık Kemal University School of Medicine, Tekirdağ 59030, Turkey; narmina.khanmammadova@hs.uci.edu; 2 Center for Applied Data Science, TED University, Ankara 06420, Turkey; taha.sarnic@tedu.edu.tr (T.E.S.); utku.turkmen@tedu.edu.tr (İ.U.T.); 3 Department of Pediatric Surgery, Ankara University School of Medicine, Ankara 06100, Turkey; uates@ankara.edu.tr (U.A.); ggollu@ankara.edu.tr (G.G.B.); mcakmak@ankara.edu.tr (A.M.Ç.); 4 Department of Pediatric Surgery, İzmir Katip Çelebi University School of Medicine, İzmir 35620, Turkey; mustafaonur.oztan@ikc.edu.tr; 5 Department of Pediatric Surgery, Selçuk University School of Medicine, Konya 42130, Turkey; tamersekmenli@selcuk.edu.tr; 6 Department of Pediatric Surgery, Yozgat State Hospital, Yozgat 66100, Turkey; nfaras@erciyes.edu.tr; 7 Department of Pediatric Surgery, Diyarbakır Gazi Yaşargil Training and Research Hospital, Diyarbakır 21090, Turkey; tulin.oztas@sbu.edu.tr (T.Ö.); salim.bilici@sbu.edu.tr (S.B.); 8 Department of Pediatric Surgery, Gazi University School of Medicine, Ankara 06500, Turkey; aliy@gazi.edu.tr (A.Y.); murato@gazi.edu.tr (M.Ö.); denizg@gazi.edu.tr (D.G.); ramazank@gazi.edu.tr (R.K.); 9 Department of Pediatric Surgery, Karadeniz Teknik University School of Medicine, Trabzon 61080, Turkey; sonayyalcin@ktu.edu.tr (H.S.Y.C.); mustafaimamoglu@ktu.edu.tr (M.İ.); haluksarihan@ktu.edu.tr (H.S.); 10 Department of Pediatric Surgery, Pamukkale University School of Medicine, Denizli 20160, Turkey; ouzunlu@pau.edu.tr; 11 Department of Pediatric Surgery, Okmeydanı Prof. Dr. Cemil Taşçıoğlu State Hospital, İstanbul 34384, Turkey; aliye.kandirici@sbu.edu.tr; 12 Department of Pediatric Surgery, Muğla Sıtkı Koçman University School of Medicine, Muğla 48000, Turkey; erturknazile@mu.edu.tr (N.E.); suzenalev@mu.edu.tr (A.S.); karakuscuneyt@mu.edu.tr (S.C.K.); 13 Department of Pediatric Surgery, Biruni University School of Medicine, İstanbul 34010, Turkey; fatihakova@biruni.edu.tr (F.A.); mehmetpasaoglu@biruni.edu.tr (M.P.); 14 Department of Pediatric Surgery, Amerikan Hospital, İstanbul 34365, Turkey; egemene@amerikanhastanesi.org

**Keywords:** appendicitis, pediatrics, machine learning, diagnosis, random forest, clinical decision support

## Abstract

**Background**: Accurate diagnosis of acute appendicitis in children remains challenging due to variable presentations and limitations of existing clinical scoring systems. While machine learning (ML) offers a promising approach to enhance diagnostic precision, most prior studies have been limited by small sample sizes, single-center data, or a lack of external validation. **Methods**: This prospective, multicenter study included 8586 pediatric patients to develop a machine learning-based diagnostic model using routinely available clinical and hematological parameters. A separate, prospectively collected external validation cohort of 3000 patients was used to assess model performance. The Random Forest algorithm was selected based on its superior performance during model comparison. Diagnostic accuracy, sensitivity, specificity, Area Under Curve (AUC), and calibration metrics were evaluated and compared with traditional scoring systems such as Pediatric Appendicitis Score (PAS), Alvarado, and Appendicitis Inflammatory Response Score (AIRS). **Results**: The ML model outperformed traditional clinical scores in both development and validation cohorts. In the external validation set, the Random Forest model achieved an AUC of 0.996, accuracy of 0.992, sensitivity of 0.998, and specificity of 0.993. Feature-importance analysis identified white blood cell count, red blood cell count, and mean platelet volume as key predictors. **Conclusions**: This large, prospectively validated study demonstrates that a machine learning-based scoring system using commonly accessible data can significantly improve the diagnosis of pediatric appendicitis. The model offers high accuracy and clinical interpretability and has the potential to reduce diagnostic delays and unnecessary imaging.

## 1. Introduction

Acute appendicitis is one of the most common surgical emergencies in childhood, accounting for a significant proportion of abdominal pain cases presented to pediatric emergency departments worldwide. The annual incidence of appendicitis is estimated at approximately 233 per 100,000 individuals, with a lifetime risk ranging from 6.7% to 8.6% [1]. In children, especially those under the age of five, the diagnosis is particularly challenging due to atypical presentations and communication barriers. Despite being a well-recognized entity, timely and accurate diagnosis of appendicitis remains a critical concern, as delays can result in complications such as perforation, abscess formation, and peritonitis [1,2]. Traditional diagnostic tools include a combination of clinical examination, laboratory investigations, and imaging modalities such as ultrasonography or computed tomography (CT). However, reliance on imaging is not without drawbacks—exposure to radiation, operator dependency, and cost being notable limitations [3,4]. Clinical scoring systems such as the Alvarado Score, Pediatric Appendicitis Score (PAS), Appendicitis Inflammatory Response Score (AIRS), and Shera Score have been widely used to aid decision-making but often lack sufficient sensitivity or specificity when applied across varied pediatric populations [1,5,6]. The reported negative appendectomy rates remain as high as 20% in some series, and up to 30–40% of children still present with perforated appendicitis at the time of diagnosis [1,7]. These statistics highlight the persistent need for improved, objective, and reproducible diagnostic methods that can be integrated into real-world clinical workflows.

Recent advances in artificial intelligence (AI), particularly in machine learning (ML), offer promising solutions to address diagnostic uncertainty in acute care settings. Machine learning algorithms excel at detecting complex patterns in large, multidimensional datasets—capabilities that are increasingly leveraged in medical diagnostics, prognosis prediction, image interpretation, and clinical decision support [8]. In the context of emergency medicine, ML has demonstrated utility in diverse domains, including the triage of chest pain, early sepsis detection, and risk stratification in trauma. Applied to appendicitis, ML models have been developed to classify patients based on clinical and biochemical features with encouraging performance metrics, often surpassing traditional scoring systems in sensitivity and specificity [2,9,10]. However, most of these models are limited by small sample sizes, homogenous data sources, or a lack of external validation. Furthermore, there is a growing demand for interpretable, transparent, and clinically deployable ML solutions, especially in pediatric populations where diagnostic thresholds and expressions differ significantly from adults. The integration of AI-based tools into routine practice necessitates rigorous validation across multiple centers and demographic settings to ensure their generalizability and safety.

Complete blood count (CBC) is one of the most ordered, rapid, and cost-effective laboratory tests in emergency settings. Several parameters within the CBC—such as white blood cell count (WBC), neutrophil-to-lymphocyte ratio (NLR), and mean platelet volume (MPV)—have been studied as potential biomarkers for diagnosing appendicitis and its complications. Studies have shown that elevated WBC and NLR are associated with both uncomplicated and perforated appendicitis, while increased MPV has been suggested as a predictor of perforation severity [11,12]. For example, a study evaluating the predictive role of MPV and NLR in appendicitis found that both markers were significantly higher in patients with perforated appendicitis compared to those with phlegmonous or localized peritonitis. Similarly, other research has demonstrated that NLR values above specific cut-offs can effectively differentiate between complicated and uncomplicated appendicitis. Beyond appendicitis, CBC-derived parameters have also been utilized in the diagnostic stratification of diseases such as COVID-19, myocardial infarction, and various malignancies, emphasizing their versatility and clinical value [13,14,15]. Nevertheless, the interpretation of CBC components is often influenced by age, comorbid conditions, and inter-individual variability, underscoring the need for models that can synthesize these variables into reliable diagnostic tools.

Despite the availability of multiple scoring systems and laboratory markers, diagnostic errors and delays in pediatric appendicitis persist. Many existing tools have limited generalizability, are not tailored to pediatric populations, or depend heavily on imaging studies, which may not always be available or advisable. Moreover, machine learning studies in this domain often suffer from methodological weaknesses, including overfitting, lack of calibration analysis, and absence of head-to-head comparisons with established clinical tools [10,16,17]. Importantly, there remains a gap in the literature for validated, externally tested ML-based scoring systems that utilize routinely available clinical and laboratory data to support rapid, non-invasive diagnosis in diverse pediatric populations. In this study, we aimed to address these limitations by externally validating a machine learning-derived scoring system for pediatric appendicitis using a large, multicenter dataset. We hypothesized that by prospectively addressing the limitations of our previously developed and published model and enhancing its predictive performance using a larger and more diverse dataset, the refined machine learning algorithm would demonstrate superior diagnostic accuracy compared to conventional scoring systems and serve as a clinically reliable decision support tool in the evaluation of suspected pediatric appendicitis [18].

## 2. Materials and Methods

### 2.1. Study Design and Setting

From 1 January to 31 December 2019, after receiving Institutional Review Board approval (2018.322.IRB2.048) and in accordance with the Declaration of Helsinki, a prospective, multicenter, national cohort study was conducted in 13 tertiary pediatric hospitals across Türkiye. The study aimed to improve and externally validate a previously published machine learning-based scoring system that was originally developed using retrospective data from five centers. The current study adhered to STROBE criteria and was designed to prospectively capture clinical, laboratory and radiological data from pediatric patients presenting with abdominal pain to emergency departments. Data collection was performed in real-time by attending physicians who supervised case recruitment, ensuring the accuracy and integrity of the records. Participation in the study was voluntary and uncompensated. Patients with known comorbidities or who had received antibiotics within the preceding week were excluded from enrollment. All clinical management decisions were made independently by treating physicians without influence from the study team. Final diagnoses were established through histopathological examination of surgical specimens, which served as the gold standard for both confirming appendicitis and classifying its severity. Cases were categorized as non-complicated appendicitis if inflammation was limited, and as complicated appendicitis if gangrenous or perforated features were identified on histopathology.

### 2.2. Participants

As part of the study protocol, a total of 8586 pediatric patients who presented to emergency departments with abdominal pain were enrolled for model development. Data were collected prospectively at the time of presentation using a standardized digital data entry system designed for uniform and structured input across centers. The dataset included 63 variables comprising demographics, clinical history, physical examination findings, CBC parameters, imaging results, intraoperative and pathology data, and clinical scoring systems such as Alvarado, PAS, AIR, RIPASA, and Lintula. Observations with missing essential variables or biologically implausible outliers (e.g., extreme red cell distribution width values) were excluded, resulting in a cleaned dataset of 8586 observations used to develop the machine learning-based appendicitis prediction model.

Following model development, a separate group of 3036 pediatric patients from the same participating centers was used for external validation. These patients were also evaluated prospectively, independent of the training dataset. Inclusion criteria for both phases were as follows: (1) age between 3 and 18 years, (2) clinical suspicion of acute appendicitis, and (3) availability of complete preoperative clinical and CBC data. Patients were excluded if they had known comorbidities, had taken antibiotics within the previous week, or lacked definitive histopathological results. Clinical management decisions were made independently by healthcare providers. Final diagnoses were determined by histopathological examination, which also served as the reference standard for classification into non-complicated or complicated appendicitis.

### 2.3. Outcome Definition

The primary outcome was the accurate diagnosis of acute appendicitis, confirmed by histopathological examination of the surgical specimen. Patients were categorized as having acute appendicitis or not based on histological findings. A secondary classification stratified cases into uncomplicated (e.g., phlegmonous appendicitis) and complicated appendicitis (e.g., perforation, gangrene, abscess formation). Negative appendectomy was defined as the removal of a macroscopically normal appendix in the absence of histological signs of inflammation. These definitions were applied consistently across all centers.

### 2.4. Data Collection and Variables

All clinical and laboratory variables were extracted from electronic health records using a standardized data abstraction protocol. Demographic variables included age and sex. Clinical presentation data encompassed symptoms such as migratory right lower quadrant (RLQ) pain, nausea/vomiting, anorexia, fever, and RLQ tenderness. Laboratory parameters included CBC-derived indices such as white blood cell (WBC) count, neutrophil count, lymphocyte count, platelet count, mean platelet volume (MPV), red cell distribution width (RDW), and hematocrit. From these, derived ratios such as the neutrophil-to-lymphocyte ratio (NLR) and platelet-to-lymphocyte ratio (PLR) were calculated. Laboratory data were collected at the time of presentation, prior to any surgical intervention or antibiotic administration. Variables with more than 15% missing data were excluded, and the remaining missing values were imputed using the median imputation strategy. All numerical variables were standardized to z-scores to facilitate model performance across different centers.

### 2.5. Model Development and Validation

The original scoring system was developed using supervised machine learning techniques on a cohort of 8586 pediatric patients. A total of 71 different models were generated and compared using performance metrics such as accuracy, sensitivity, specificity, and AUC. The final model selected was a random forest classifier, which demonstrated the highest diagnostic accuracy and generalizability. For the current validation, the model was applied without retraining to an independent multicenter dataset of 3036 pediatric patients, to assess its external validity. Model inputs consisted of the same 12 standardized clinical and laboratory variables used in the original derivation cohort. The performance of the model was evaluated in terms of its ability to distinguish between appendicitis and non-appendicitis cases, as well as between complicated and uncomplicated appendicitis.

### 2.6. Comparison with Existing Scores

To contextualize the performance of the machine learning-based score, the same validation dataset was analyzed using three commonly used clinical scoring systems: the Alvarado score, the PAS, and the AIRS. Scores were calculated using available clinical and laboratory data, and their diagnostic accuracy was evaluated using ROC analysis. Each score’s performance was compared to that of the machine learning model using AUC, sensitivity, specificity, positive predictive value (PPV), and negative predictive value (NPV). Comparative analysis was performed to assess whether the new model provided incremental value over traditional scoring systems.

### 2.7. Statistical Analysis

All statistical analyses were performed using Python 2.7.16. The normality of continuous variables was assessed using the Kolmogorov–Smirnov test. Continuous variables were presented as means and standard deviations, while categorical variables were presented as frequencies and percentages. Model performance was evaluated using metrics including sensitivity, specificity, PPV, NPV, accuracy, and AUC. ROC curves were plotted. Feature importance was analyzed via recursive feature elimination and decision tree visualization. To prevent overfitting, five-fold cross-validation was applied. A *p*-value of <0.05 was considered statistically significant for all hypothesis tests.

## 3. Results

### 3.1. Patient Characteristic

The study cohort comprised 8586 pediatric patients for model development, with a higher rate of male patients (n = 6816, 79.39%). The mean age was 7.5 ± 5.37 years. Despite the dominance of the male gender, no statistically significant correlation was observed between the patient’s diagnosis and gender (*p* = 0.54). There were 3954 (46.05%) patients diagnosed with appendicitis, of which 3658 (92.51%) were non-complicated cases.

The most common presenting symptoms were abdominal pain, vomiting, nausea, and fever. Among all patients, 4462 (51.96%) had an X-ray scan, and 5071 (59.06%) underwent ultrasonography (USG). Appendicolith was found in 3 patients (0.06%) via X-ray, free air in 1 (0.02%), and air-fluid levels in 36 (0.8%). Among patients who underwent USG, 686 (13.53%) were diagnosed with appendicitis.

The external validation dataset included 3036 patients, with 69.50% male and a mean age of 11.29 ± 3.99 years. The demographic features between the appendicitis and non-appendicitis groups were similar. Among these patients, 94.37% were diagnosed with appendicitis, with 42.40% being non-complicated and 51.97% complicated.

### 3.2. Model Performance

During model development, white blood cell variables showed no statistically significant difference between appendicitis and non-appendicitis groups (*p* > 0.05), although higher median values were observed in the appendicitis group (Table 1). Patients’ visualizations of the parameters (e.g., diagonal density plots, scatter plots) revealed overlapping distributions between groups, limiting clear separability. However, trends such as higher MPV and lower PLT in appendicitis patients, as well as extreme high/low MCV values and concentrated high MCHC in the appendicitis group, were observed.

While several models were trained and evaluated, the Random Forest (RF) model demonstrated the highest performance in predicting the presence of appendicitis. In the external validation dataset, the RF model again achieved outstanding results with an AUC of 0.996, accuracy of 0.992, sensitivity of 0.998, and specificity of 0.993 for appendicitis diagnosis (Table 2).

For classification of appendicitis as complicated vs. non-complicated, the RF model maintained high performance with an AUC of 0.995, accuracy of 0.992, sensitivity of 0.993, and specificity of 0.991 (Table 3).

### 3.3. Comparison with Traditional Scoring Systems

After identifying the most predictive variables, the dataset was analyzed using multiple existing scoring systems including PAS, AIRS, and Alvarado (Table 4). While these traditional systems showed high specificity, their sensitivity and accuracy were considerably lower. Among them, PAS had the highest success rate. In contrast, Random Forest achieved over 95% success across all performance metrics in both development and validation datasets Table 5).

### 3.4. Calibration and Feature Importance

Although classical statistical significance was limited for many individual variables, several feature trends were observed in model-based and visualization analyses. Density plots indicated weak individual separability, but the machine learning model was able to leverage complex feature interactions. Visual analysis and ranking of predictors in the RF model highlighted the importance of WBC, MPV, PLT, MCHC, and MCV variables for appendicitis prediction. Detailed rankings are provided in the Supplementary Materials.

## 4. Discussion

In this multicenter, prospective cohort study, we developed and validated a machine learning-based scoring system to improve the diagnostic accuracy of acute appendicitis in pediatric patients. Using a dataset of 8586 patients from 13 tertiary pediatric centers in Türkiye, we trained several machine learning models and identified the RF algorithm as the most effective in distinguishing appendicitis cases. External validation on a separate cohort of 3036 patients confirmed the model’s robust performance, achieving an AUC of 0.996, accuracy of 0.992, sensitivity of 0.998, and a specificity of 0.993.

The results clearly demonstrate the model’s capacity to outperform traditional scoring systems such as PAS, AIRS, and Alvarado. While these clinical tools have been widely used, our findings revealed that they suffered from relatively lower sensitivity and overall accuracy in both model development and validation phases [19,20]. In contrast, the RF model maintained high performance across all evaluation metrics. This reinforces the potential of machine learning-based models to enhance diagnostic precision in conditions where traditional tools may lack generalizability or are insufficiently sensitive.

Feature analysis revealed that while many classical hematological parameters such as white blood cell count and neutrophil percentage showed overlapping distributions between appendicitis and non-appendicitis groups, the machine learning model successfully integrated subtle variations across multiple features. Notably, MPV, PLT, MCHC, and MCV contributed significantly to the model’s predictions. These findings align with prior literature suggesting the diagnostic value of inflammatory and red blood cell indices in appendicitis, although their standalone clinical utility remains limited due to inter-individual variability.

In recent years, several studies have implemented ML techniques to support the diagnosis of acute appendicitis, particularly in pediatric populations [6,19,21,22]. A 2023 systematic review by Lam et al. emphasized that although ML models such as random forest, SVM, and deep learning architectures have demonstrated promising results with AUC values commonly exceeding 0.90, most studies suffer from key limitations such as small sample size, lack of external validation, and inconsistent outcome definitions [10]. For instance, Tamyalew et al. developed an X-ray-based ML model for large bowel obstruction classification using deep convolutional neural networks but lacked a structured clinical input dataset, which limits its comparability to symptom- and CBC-based approaches [23]. Similarly, Yu et al. demonstrated limited standalone performance for CRP, WBC, and procalcitonin in appendicitis, supporting the integration of such features into multidimensional ML models [24].

Notably, recent individual studies have explored ML applications in pediatric appendicitis with varying input features (CBC components, symptoms, ultrasound findings) [20,25]. However, most of these models were evaluated on retrospective data with fewer than 1000 patients and often used single-center records [21]. In contrast, the present study includes over 8500 patients for model development and 3000 for prospective validation across 13 tertiary centers, making it one of the largest and most rigorously validated appendicitis prediction models published to date. Moreover, while previously reported AUCs typically range between 0.85 and 0.94, the random forest model in this study achieved an AUC of 0.996, outperforming even the most recent comparable models in both diagnosis and severity classification. Unlike earlier works that focused narrowly on appendicitis vs. non-appendicitis, the present model also reliably differentiates between complicated and non-complicated appendicitis—a clinical distinction of increasing importance given the growing interest in non-operative management.

The external validation results are particularly noteworthy. Despite being applied to a distinct patient cohort from the same centers and timeframe, the model preserved its diagnostic strength, confirming its consistency and real-world applicability. This is crucial in pediatric populations where atypical symptom presentations and communication limitations frequently lead to diagnostic delays or unnecessary imaging and surgery.

A further strength of the study lies in its scale and design. With over 11,000 patients evaluated across development and validation phases, this is one of the largest prospective pediatric appendicitis modeling efforts to date. Additionally, all data were collected prospectively using a standardized digital infrastructure, minimizing documentation bias and maximizing uniformity. Histopathology was used as the definitive diagnostic standard, further strengthening the reliability of outcomes.

Nevertheless, several limitations must be acknowledged. First, although the external validation set was prospectively collected, it originated from the same institutional network within the same year, which may limit generalizability to other healthcare systems. Second, we did not assess the model’s impact on clinical decision-making, resource utilization, or patient outcomes in real time. These would require prospective interventional studies. Third, while model performance was excellent, the black-box nature of machine learning models, including Random Forest, may limit clinician interpretability, even with feature-importance analysis.

In conclusion, this study demonstrates that a robust, prospectively validated machine learning-based model can significantly improve diagnostic accuracy for pediatric appendicitis. The RF model outperformed existing clinical scores and maintained consistent performance in an external cohort. With further validation and implementation studies, such tools may enhance early diagnosis, reduce reliance on imaging, and improve outcomes in pediatric emergency care.

## Figures and Tables

**Table 1:** Comparison of complete blood count parameters between groups.

		Appendicitis	Non-Appendicitis	*p*
Red Blood Cell Variables	Hgb	12.6 ± 1.40	12.6 ± 1.41	0.9087
	Htc	37.5 ± 3.92	37.6 ± 3.94	0.3276
	RDW	13.1 ± 1.85	13.2 ± 1.57	0.0117
	MCV	83.8 ± 8.96	78.5 ± 3.54	0.0000
	MCHC	33.6 ± 1.37	33.1 ± 1.53	0.0000
White Blood Cell Variables	WBC	12,315 ± 5460	12,050 ± 5400	0.1107
	Lymphocyte	2710 ± 2037	2690 ± 2090	0.8771
	Neutrophil	7220 ± 5732	6895 ± 5609	0.2133
	NLR	2.47 ± 6.79	2.34 ± 6.77	0.4547
Thrombosis Variables	Platelet	295,000 ± 93,305	310,000 ± 92,694	0.0000
	MPV	7.9 ± 1.46	6.84 ± 1.44	0.0000
	PDW	18.9 ± 16.0	18.7 ± 5.02	0.0000

Hgb: Hemoglobin, Htc: Hematocrit, RDW: Red Cell Distribution Width, MCV: Mean Corpuscular Volume, MCHC: Mean Corpuscular Hemoglobin Concentration, WBC: White Blood Cell Count, Lymphocyte: Lymphocyte Count, Neutrophil: Neutrophil Count, NLR: Neutrophil-to-Lymphocyte Ratio, Platelet: Platelet Count, MPV: Mean Platelet Volume, PDW: Platelet Distribution Width.

**Table 2:** The performance parameters of scoring systems and the proposed models to diagnose appendicitis in the new dataset.

Scoring System/Model	AUC	Accuracy	Sensitivity	Specificity
Alvarado	0.632	0.744	0.326	0.852
Lintula	0.816	0.847	0.650	0.892
PAS	0.926	0.843	0.872	0.858
RIPASA	0.683	0.758	0.321	0.795
LR	0.986	0.975	0.988	0.972
KNN	0.988	0.979	0.997	0.963
SVM	0.982	0.983	0.995	0.973
CART	0.994	0.976	0.997	0.967
RF	0.996	0.992	0.998	0.993

PAS: Pediatric Appendicitis Score; RIPASA: Raja Isteri Pengiran Anak Saleha Appendicitis; LR: Logistic Regression; KNN: k^th^ Nearest Neighbor; SVM: Support Vector Machine; CART: Classification and Regression Tree; RF: Random Forest; AUC: Area Under the Receiver Operating Characteristic Curve.

**Table 3:** The performance parameters of models to diagnose appendicitis.

Model	AUC	Accuracy	Sensitivity	Specificity
KNN	0.979	0.946	0.976	0.975
SVM	0.994	0.941	0.992	0.976
CART	0.987	0.952	0.913	0.962
RF	0.995	0.992	0.993	0.991

LR: Logistic Regression; KNN: kth Nearest Neighbor; SVM: Support Vector Machine; CART: Classification and Regression Tree; RF: Random Forest; AUC: Area Under the Receiver Operating Characteristic Curve.

**Table 4:** The performance parameters of scoring systems and the proposed models to diagnose appendicitis.

Scoring System/Model	AUC	Accuracy	Sensitivity	Specificity
Alvarado	0.728	0.871	0.489	0.967
Lintula	0.863	0.925	0.760	0.966
PAS	0.944	0.953	0.927	0.960
RIPASA	0.714	0.865	0.469	0.967
LR	0.968	0.962	0.982	0.948
KNN	0.977	0.969	0.997	0.948
SVM	0.974	0.967	0.997	0.946
CART	0.982	0.968	0.994	0.949
RF	0.984	0.968	0.991	0.951

PAS: Pediatric Appendicitis Score; RIPASA: Raja Isteri Pengiran Anak Saleha Appendicitis; LR: Logistic Regression; KNN: kth Nearest Neighbor; SVM: Support Vector Machine; CART: Classification and Regression Tree; RF: Random Forest; AUC: Area Under the Receiver Operating Characteristic Curve.

**Table 5:** The performance parameters of models to classify appendicitis.

Model	AUC (CV)	AUC	Accuracy	Sensitivity	Specificity
KNN	0.962	0.973	0.913	0.958	0.911
SVM	0.968	0.970	0.936	1.000	0.933
CART	0.956	0.971	0.939	0.833	0.943
RF	0.968	0.973	0.925	0.875	0.927

LR: Logistic Regression; KNN: kth Nearest Neighbor; SVM: Support Vector Machine; CART: Classification and Regression Tree; RF: Random Forest; AUC: Area Under the Receiver Operating Characteristic Curve; CV: Cross Validation (5×).

## Data Availability

The data supporting the findings of this study are not publicly available due to privacy and ethical restrictions. However, the algorithm may be made available by the corresponding author upon reasonable request for research purposes.

## Supplementary Materials

The following supporting information can be downloaded at: https://www.mdpi.com/article/10.3390/children12070937/s1.

## Abbreviations

The following abbreviations are used in this manuscript:

AUC	Area Under the Curve
AIRS	Appendicitis Inflammatory Response Score
CART	Classification and Regression Tree
CBC	Complete Blood Count
CT	Computed Tomography
LR	Logistic Regression
KNN	k-th Nearest Neighbor
ML	Machine Learning
MPV	Mean Platelet Volume
MCV	Mean Corpuscular Volume
MCHC	Mean Corpuscular Hemoglobin Concentration
NLR	Neutrophil-to-Lymphocyte Ratio
PAS	Pediatric Appendicitis Score
PLT	Platelet Count
PLR	Platelet-to-Lymphocyte Ratio
RF	Random Forest
RIPASA	Raja Isteri Pengiran Anak Saleha Appendicitis Score
ROC	Receiver Operating Characteristic
SVM	Support Vector Machine
USG	Ultrasonography
WBC	White Blood Cell Count
