# Supplementary material for: Diagnostic Accuracy of a Machine Learning-Derived Appendicitis Score in Children: A Multicenter Validation Study
Source: Children (Basel). 2025 Jul 16;12(7):937. doi: 10.3390/children12070937 (PMC12293784; doi:10.3390/children12070937)
Supplement: Supplementary file 1 [file children-12-00937-s001.zip › children-3679230-supplementary.pdf]

**Prediction of Appendicitis**

<u>White blood cell</u>

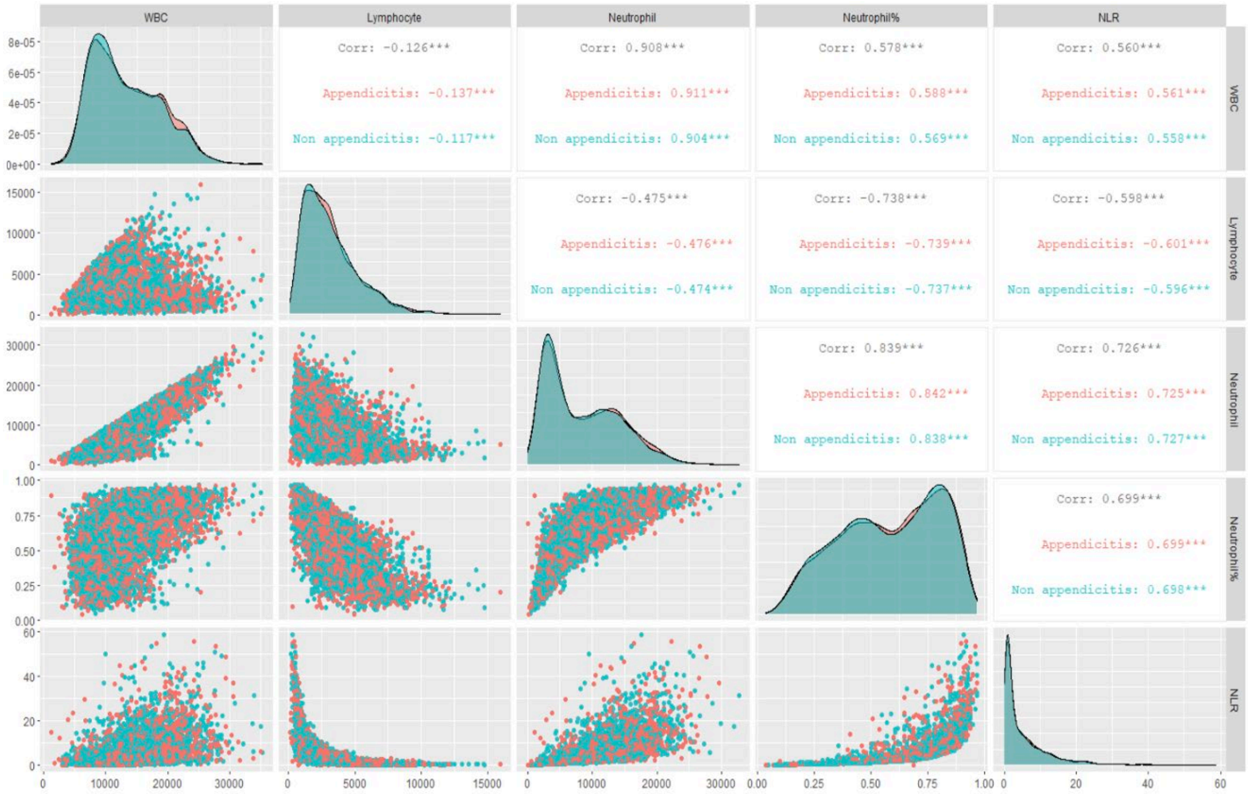


| White Blood Cell Variables | Appendicitis | | Non-appendicitis | | $U^a$ | $p$ |
|---|---|---|---|---|---|---|
| | *MD* | *SD* | *MD* | *SD* | | |
| WBC | 12,315 | 5,459.989 | 12,050 | 5,400 | 9,340,056 | 0.1107 |
| Lymphocyte (µL) | 2,710 | 2,037 | 2,690 | 2,090 | 9,139,758 | 0.8771 |
| Neutrophil (µL) | 7,220 | 5,732 | 6,895 | 5,609 | 9,299,950 | 0.2133 |
| Neutrophil (%) | 0.61 | 0.220 | 0.6 | 0.220 | 9,242,245 | 0.4589 |
| NLR | 2.47 | 6.79 | 2.34 | 6.77 | 9,243,050 | 0.4547 |

[a]Non-parametric Mann-Whitney U test statistics $^{***}p < 0.001$, $^{**}p < 0.01$, $^{*}p < 0.05$

Red blood cell

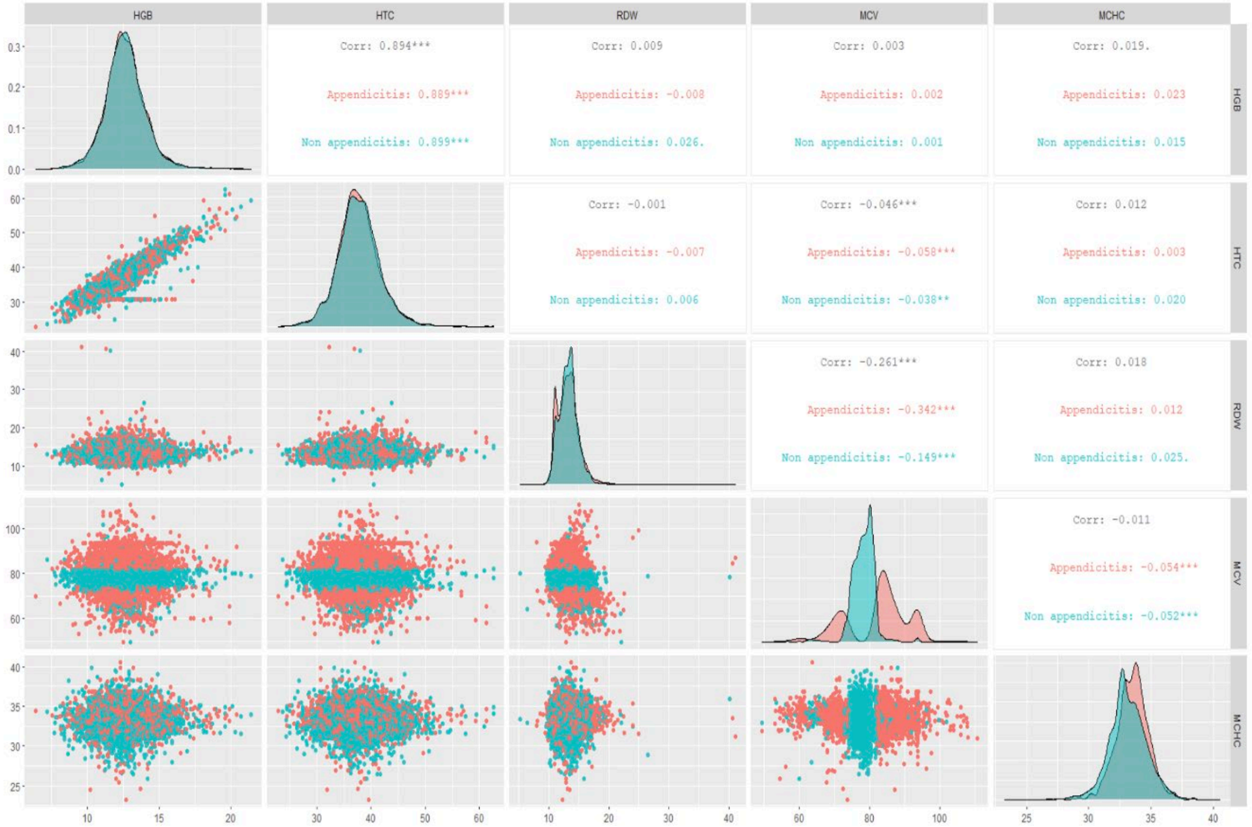


| Red Blood Cell Variables | Appendicitis | | Non-appendicitis | | *U* | *p* |
|---|---|---|---|---|---|---|
| | *MD* | *SD* | *MD* | *SD* | | |
| HGB (g/dl) | 12.6 | 1.40 | 12.6 | 1.41 | 9,170,591 | 0.9087 |
| HTC (%) | 37.5 | 3.92 | 37.6 | 3.94 | 9,045,405 | 0.3276 |
| RDW (%) | 13.1 | 1.85 | 13.2 | 1.57 | 8,869,044 | $0.01174^{*}$ |
| MCV (fL) | 83.8 | 8.96 | 78.5 | 3.54 | 13,003,485 | $0.000^{***}$ |
| MCHC (g/dl) | 33.6 | 1.37 | 33.1 | 1.53 | 10,642,974 | $0.000^{***}$ |

$^{***}p < 0.001$, $^{**}p < 0.01$, $^{*}p < 0.05$

Platelet

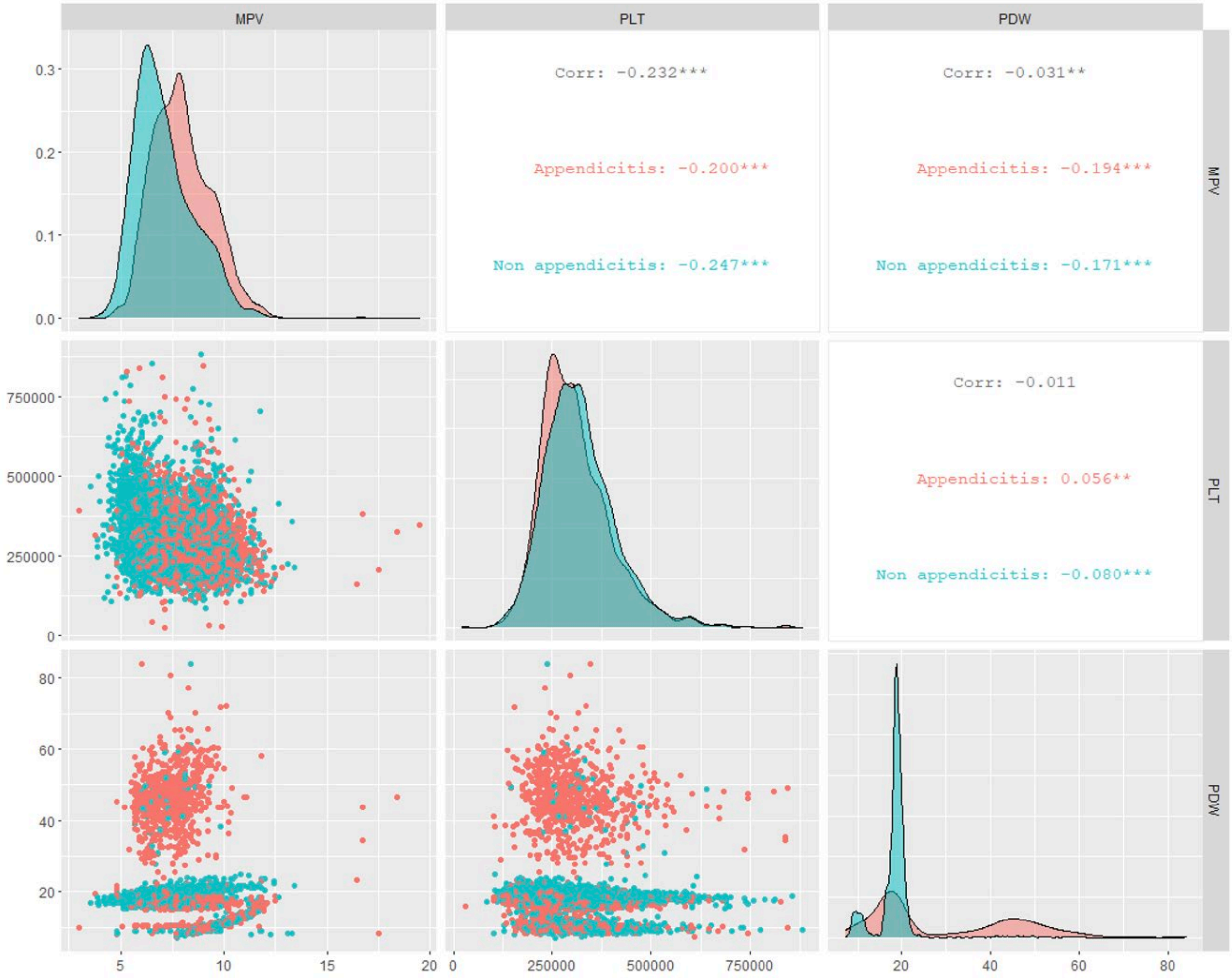


| Thrombosis Variables | Appendicitis | | Non-appendicitis | | *U* | *p* |
|---|---|---|---|---|---|---|
| | *MD* | *SD* | *MD* | *SD* | | |
| PLT | 295,000 | 93,305 | 310,000 | 92,694 | 8,345,215 | $0.000^{***}$ |
| MPV (fL) | 7.9 | 1.46 | 6.84 | 1.44 | 11,996,684 | $0.000^{***}$ |
| PDW (%) | 18.9 | 16.0 | 18.7 | 5.02 | 9,073,812 | $0.000^{***}$ |

$^{***}p < 0.001$, $^{**}p < 0.01$, $^{*}p < 0.05$

<u>CRP</u>

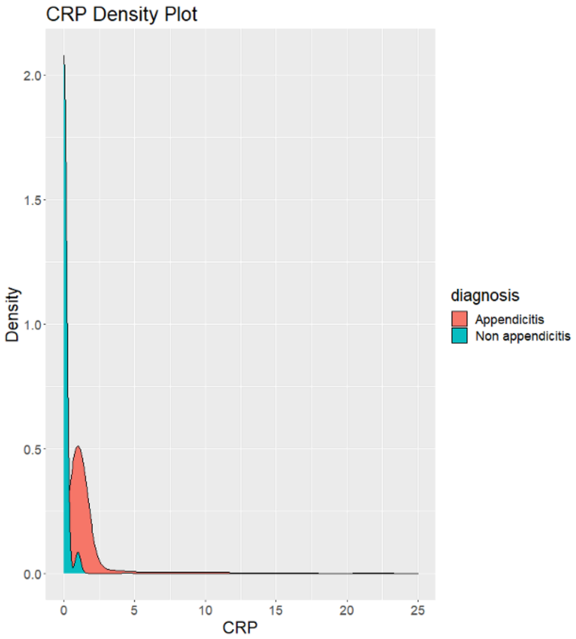


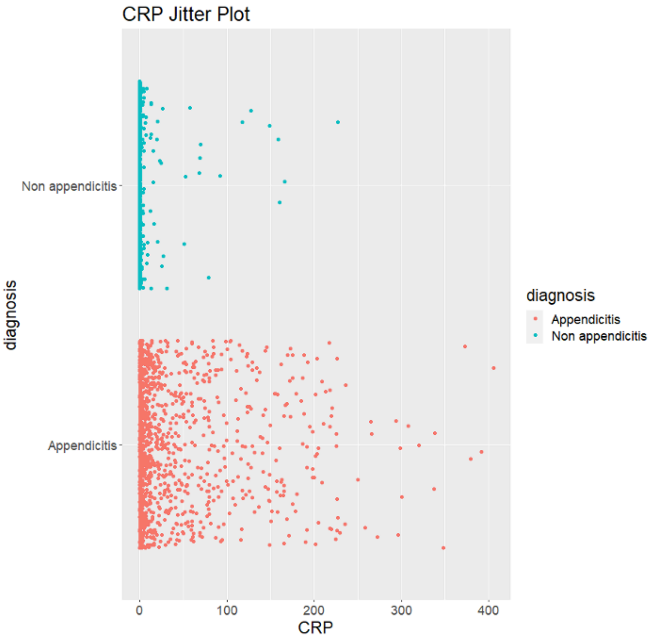


| Diagnosis | Appendicitis | | Non-appendicitis | | *U* | *p* |
|---|---|---|---|---|---|---|
| | *MD* | *SD* | *MD* | *SD* | | |
| CRP (mg/L) | 1 | 39.3 | 0 | 7.06 | 17,171,896 | $0.000^{***}$ |

$^{***}p < 0.001$, $^{**}p < 0.01$, $^{*}p < 0.05$

## Prediction of the Complex Appendicitis

<u>White blood cell</u>

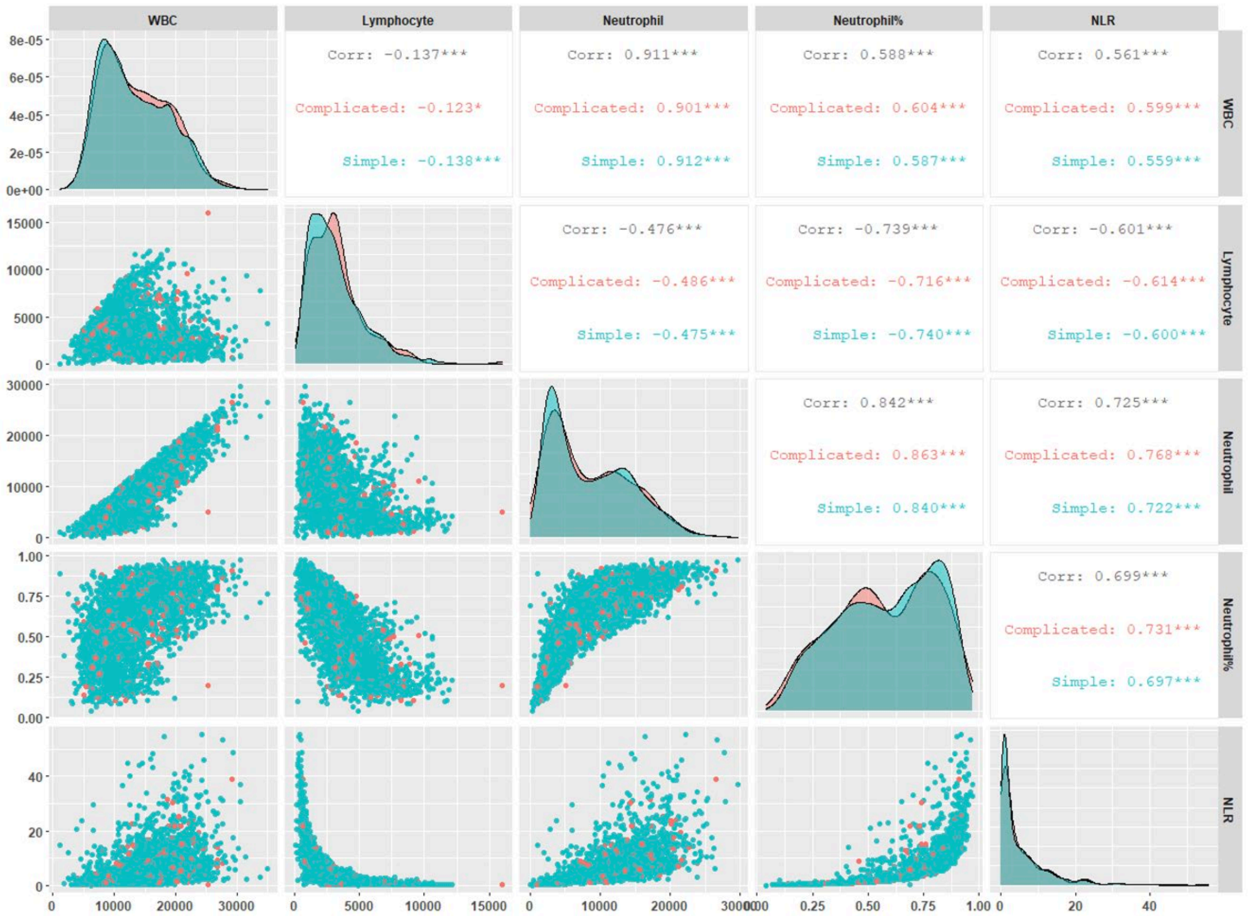


| White Blood Cell Variables | Simple | | Complex | | $U^a$ | *p* |
|---|---|---|---|---|---|---|
| | *MD* | *SD* | *MD* | *SD* | | |
| WBC | 12,248 | 5,544 | 12,959 | 5,328 | 562,418 | 0.2655 |
| Lymphocyte (μL) | 2,690 | 2,035 | 2,905 | 2,059 | 563,966 | 0.2319 |
| Neutrophil (μL) | 7,255 | 5,735 | 7,055 | 5,698 | 543,447 | 0.9131 |
| Neutrophil (%) | 0.61 | 0.220 | 0.575 | 0.221 | 524,293 | 0.3656 |
| NLR | 2.51 | 6.84 | 1.98 | 6.21 | 531,681 | 0.6075 |

$^{***}p < 0.001$, $^{**}p < 0.01$, $^{*}p < 0.05$

Red blood cell

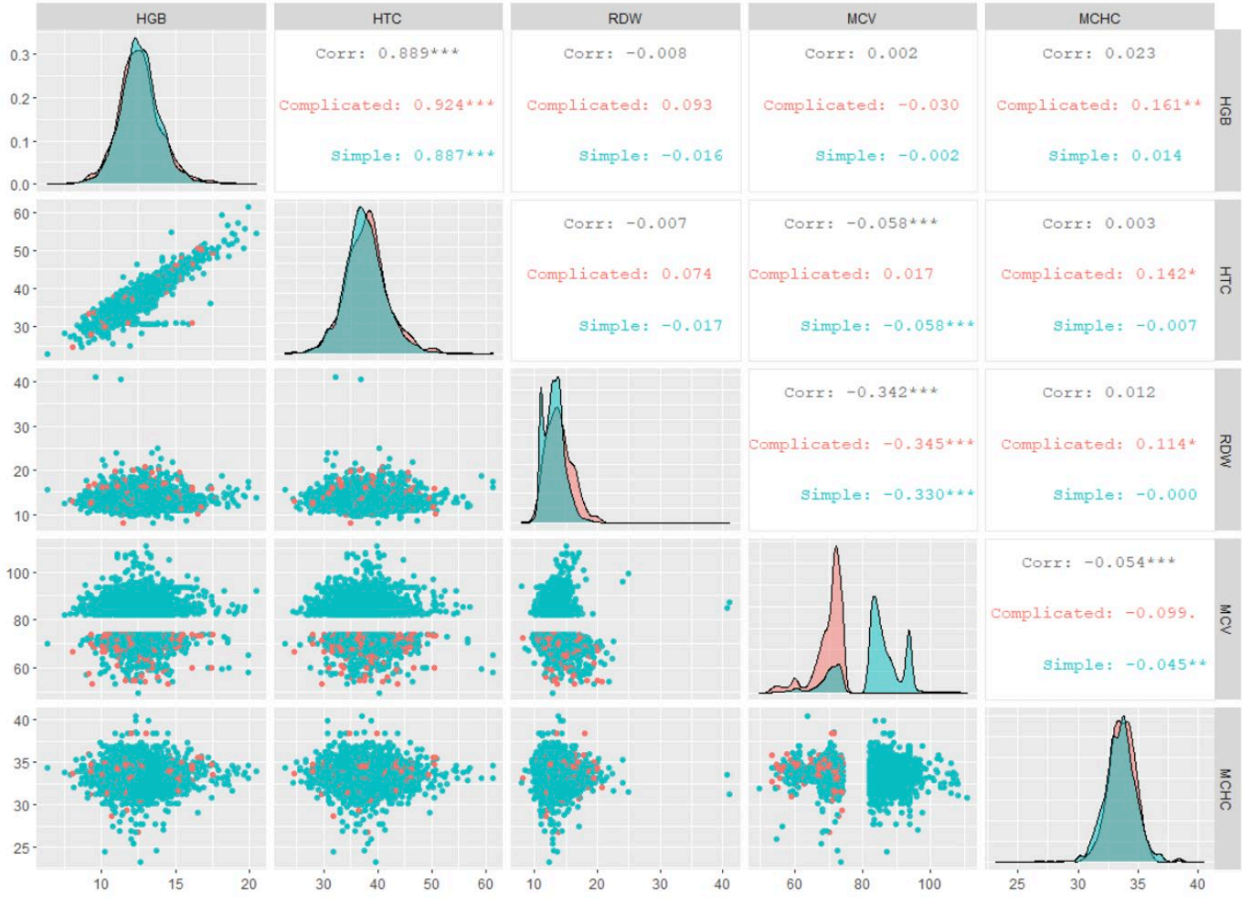


| Red Blood Cell Variables | Simple | | Complex | | *U* | *p* |
|---|---|---|---|---|---|---|
| | *MD* | *SD* | *MD* | *SD* | | |
| HGB (g/dl) | 12.6 | 1.39 | 12.5 | 1.43 | 521,362 | 0.2891 |
| HTC (%) | 37.4 | 3.92 | 38 | 3.96 | 568,684 | 0.1484 |
| RDW (%) | 13.1 | 1.82 | 13.7 | 2.08 | 664,295 | 0.000*** |
| MCV (fL) | 84.2 | 8.49 | 71.5 | 4.43 | 127,699 | 0.000*** |
| MCHC (g/dl) | 33.5 | 1.38 | 33.7 | 1.23 | 582,935 | 0.028* |

***$p < 0.001$, **$p < 0.01$, *$p < 0.05$

<u>Platelet</u>

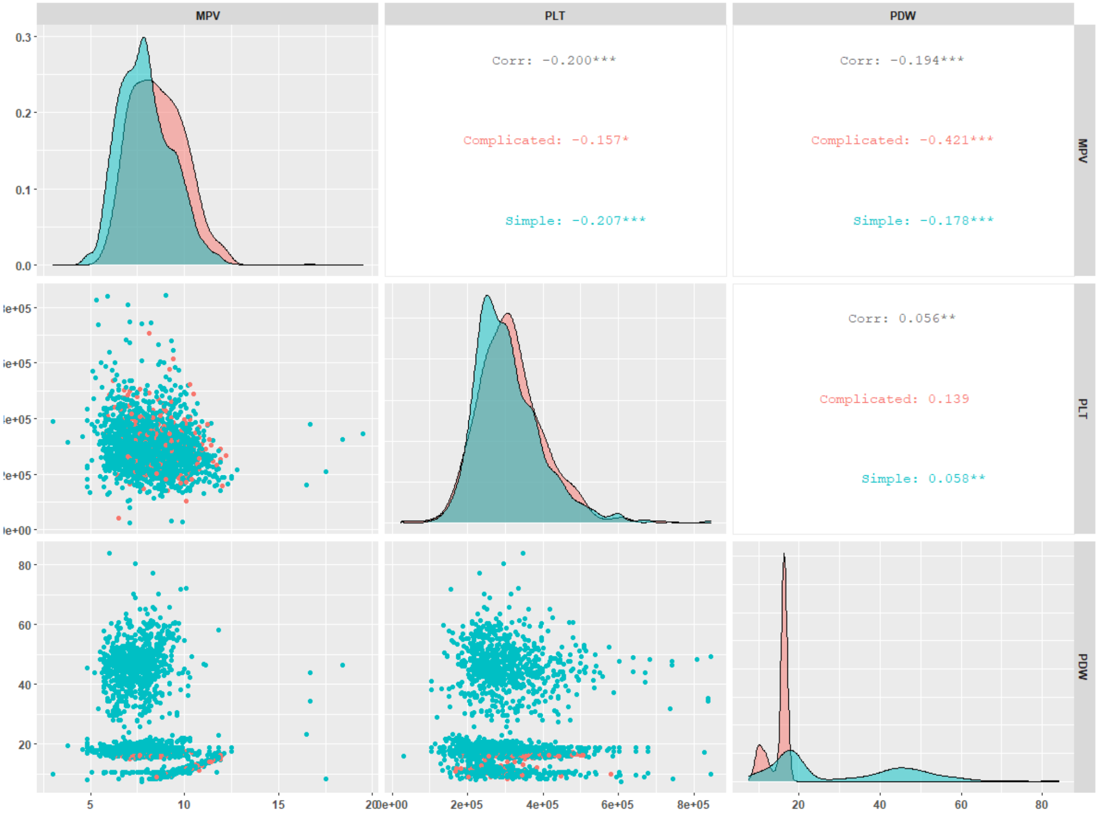


| Thrombosis Variables | Simple | | Complex | | *U* | *p* |
|---|---|---|---|---|---|---|
| | *MD* | *SD* | *MD* | *SD* | | |
| PLT | 294,000 | 93,652 | 306,000 | 88,851 | 581,479 | 0.0338* |
| MPV (fL) | 7.8 | 1.45 | 8.5 | 1.39 | 575285 | 0.000*** |
| PDW (%) | 18.9 | 16.1 | 16.1 | 2.50 | 61490 | 0.000*** |

***p < 0.001, **p < 0.01, *p < 0.05

## CRP

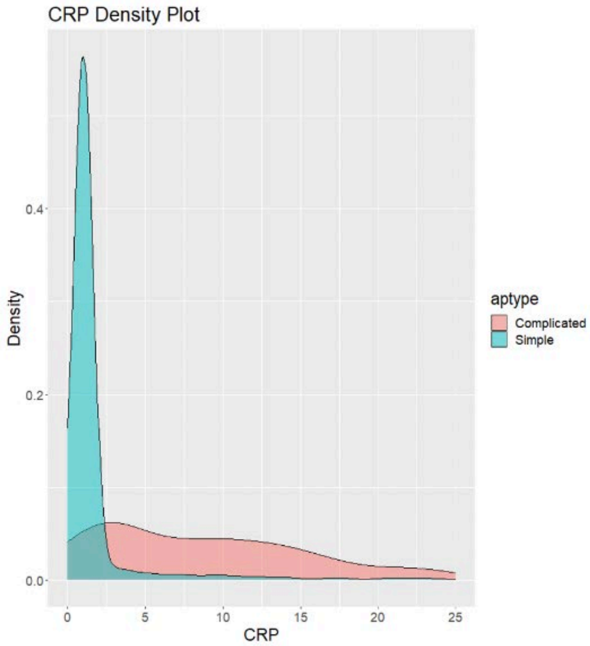


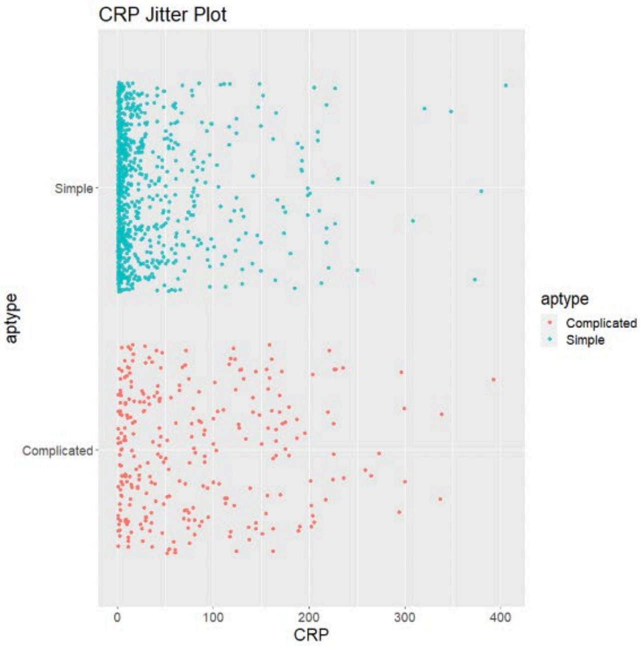


| Diagnosis | Simple | | Complex | | *U* | *p* |
|---|---|---|---|---|---|---|
| | *MD* | *SD* | *MD* | *SD* | | |
| CRP (mg/L) | 1 | 28.8 | 50.3 | 78.6 | 921,743 | 0.000*** |

***p < 0.001, **p < 0.01, *p < 0.05
